# Capsaicin pretreatment attenuates salt-sensitive hypertension by alleviating AMPK/Akt/Nrf2 pathway in hypothalamic paraventricular nucleus

**DOI:** 10.3389/fnins.2024.1416522

**Published:** 2024-05-30

**Authors:** Xiu-Yue Jia, Yu Yang, Xiao-Tao Jia, Da-Li Jiang, Li-Yan Fu, Hua Tian, Xin-Yan Yang, Xin-Yue Zhao, Kai-Li Liu, Yu-Ming Kang, Xiao-Jing Yu

**Affiliations:** ^1^Shaanxi Engineering and Research Center of Vaccine, Key Laboratory of Environment and Genes Related to Diseases of Education Ministry of China, Department of Physiology and Pathophysiology, School of Basic Medical Sciences, Xi'an Jiaotong University, Xi'an, China; ^2^Department of Physiology, Basic Medical College, Jiamusi University, Jiamusi, Heilongjiang, China; ^3^Department of Pharmacology, Basic Medical College, Jiamusi University, Jiamusi, Heilongjiang, China; ^4^Department of Neurology, The Affiliated Xi'an Central Hospital of Xi'an Jiaotong University College of Medicine, Xi'an, Shaanxi, China; ^5^Department of Urology, The First Affiliated Hospital of Xi'an Jiaotong University, Xi'an, China

**Keywords:** capsaicin, salt-sensitive hypertension, AMPK/Akt/Nrf2 pathway, hypothalamic paraventricular nucleus, inflammatory cytokines

## Abstract

**Background:**

Long term hypertension seriously promotes target organ damage in the brain and heart, and has increasingly become serious public health problem worldwide. The anti-hypertensive effects of capsaicin has been reported, however, the role and mechanism of capsaicin within the brain on salt-induced hypertension have yet to be elucidated. This study aimed to verify the hypothesis that capsaicin attenuates salt-induced hypertension via the AMPK/Akt/Nrf2 pathway in hypothalamic paraventricular nucleus (PVN).

**Methods:**

Dahl salt-sensitive (Dahl S) rats were used as animal model for the present study. Rats were randomly divided into four groups based on their dietary regimen (0.3% normal salt diet and 8% high salt diet) and treatment methods (infusion of vehicle or capsaicin in the PVN). Capsaicin was chronically administered in the PVN throughout the animal experiment phase of the study that lasted 6 weeks.

**Results:**

Our results demonstrated that PVN pretreatment with capsaicin can slow down raise of the blood pressure elevation and heart rate (HR) of Dahl S hypertensive rats given high salt diet. Interestingly, the cardiac hypertrophy was significantly improved. Furthermore, PVN pretreatment with capsaicin induced decrease in the expression of mRNA expression of NADPH oxidase-2 (NOX2), inducible nitric oxide synthase (iNOS), NOX4, p-IKKβ and proinflammatory cytokines and increase in number of positive cell level for Nrf2 and HO-1 in the PVN of Dahl S hypertensive rats. Additionally, the protein expressions of phosphatidylinositol 3-kinase (p-PI3K) and phosphorylated protein kinase-B (p-AKT) were decreased, phosphorylated adenosine monophosphate-activated protein kinase (p-AMPK) were increased after the PVN pretreatment with capsaicin.

**Conclusion:**

Capsaicin pretreatment attenuates salt-sensitive hypertension by alleviating AMPK/Akt/iNOS pathway in the PVN.

## 1 Introduction

Hypertension has been recognized as a rampant global health problem and contributes to rise of global burden of disease with increasing number of hypertensive patients (Ji et al., 2022; Burnier and Damianaki, 2023). However, the number of hypertensive patients who can control their blood pressure is still unsatisfying (Tini et al., 2023). Compelling experimental and clinical data have provided the important relation of sodium balance to blood pressure (Mattson et al., 2022; Shi et al., 2022). Individuals exhibit different blood pressure responses to salt load or salt restriction, indicating salt sensitivity issues. Salt sensitivity of blood pressure is observed in a quarter of individuals with normal blood pressure and in half of hypertensive patients (Kurtz et al., 2022). Salt sensitivity was mainly found among people with risk factor for hypertension, which include black individuals, the elderly, and first-degree relatives of hypertensive patients (Campese, 1994). This suggests that high sodium salt intake is positively associated with hypertension and constitute a significant pathogenic or aggravating factor for hypertension.

Although the specific mechanism of high salt-elevated blood pressure needs further clarifications, a growing body of evidence supports the role of sympathetic nervous system since most salt sensitive hypertensive patients and various high salt-induced hypertensive animal models exhibit sustained sympathetic nervous activity, which exacerbates the progression of hypertension (Morisawa et al., 2020; Liu et al., 2022). The presympathetic neurons located in the paraventricular nucleus of the hypothalamus (PVN), play a prominent role in the production/regulation of sympathetic vasomotor tension, affecting sympathetic overactivation and hence participating in the hypertension (Bi et al., 2022).

The pathological potential of adenosine monophosphate-activated protein kinase (AMP) activated protein kinase (AMPK) has been reported in cardiovascular and metabolic diseases, suggesting this kinase as a potential therapeutic target for the treatment and prevention of cardiovascular diseases (Wu and Zou, 2020; Rodriguez et al., 2021). AMPK is well known as sensor for cellular energy and could restore cellular metabolic homeostasis. Studies reported that the classic PI3K/Akt signaling pathway plays an important role in regulating cell metabolism, inflammation, oxidative stress, growth, and protein synthesis in mammals (Abdurahman et al., 2022; Deng and Zhou, 2023). Moreover, the regulatory effect of PI3K/Akt signaling pathway on sympathetic nerve excitability in hypertension has been reported (Zuo et al., 2021). In pathological state, Akt activation leads to that of nuclear factors-κB (NF-κB) which initiates downstream inflammatory responses. Also, PI3K pathway reportedly mediated neuroinflammation and was involved in the sympathetic hyperactivity during hypertension.

Interestingly, inhibition of PI3K/Akt pathway demonstrated therapeutic potential by targeting different processes such as inflammation, oxidative stress, and apoptosis among others, in different disease conditions such as hypertension or ischemia reperfusion injury. Thus, overexpression of central angiotensin converting enzyme 2 inhibited the PI3K/Akt pathway and neuroinflammation, and subsequently reduced peripheral sympathetic nervous activity in hypertensive rats. Moreover, eliminating PI3K activity and enhancing PI3K/Akt signal transduction reduced oxidative stress and myocardial cell apoptosis in myocardial tissue (Yu et al., 2021), regulated VSMC proliferation, migration, apoptosis and dedifferentiation, alleviated pulmonary hypertension caused by vascular remodeling, and improve vascular inflammation and oxidative stress caused by hypertension. Importantly, the activation of AMPK, which targets Akt signaling, showed a protective effect on ischemia–reperfusion injury (Tirpude et al., 2021).

The transcription factor nuclear factor erythroid 2-related factor 2 (Nrf2) regulates some antioxidant proteins. In hypertension, the downregulation of Nrf2 is crucial for disrupting central redox homeostasis and mediating sympathetic nervous activity (Gao et al., 2022). The transcription factor Nrf2 is the main regulatory factor in the cellular defense system. The activated Nrf2 translocates to the nucleus and then binds to antioxidant response elements in various detoxification and antioxidant gene promoters. In the central nervous system, Nrf2 affects sympathetic arousal and blood pressure fluctuations (Wafi, 2023). However, there is still insufficient experimental data and clinical delineating the role of AMPK/Akt/Nrf2 pathway in PVN in long-term high salt-induced hypertension in animals or patients are sparse.

China is one of the earliest countries to use chili peppers as medicine, and traditional Chinese medicine uses chili peppers to treat stomach cold, rheumatism, and other diseases. Researches showed that capsaicin, an important component of chili pepper, has anti-inflammatory, analgesic, anesthetic and detoxification effects (Zhang et al., 2022; Adhikari et al., 2023; Li et al., 2023). Its analgesic effect is equal to that of morphine, but more lasting than that of morphine. It has significant effects on postherpetic neuralgia, trigeminal neuralgia, diabetes neuralgia, rheumatoid arthritis, osteoarthritis, psoriasis, alopecia, etc. (Botz et al., 2021; Zhang et al., 2023). In addition, capsaicin can also inhibit the occurrence of malignant tumors and has special effects on treating skin diseases, weight loss, etc. (Georgescu et al., 2017; Zhang et al., 2023). Recently, it has been reported to improve blood pressure in a rat model of renovascular hypertension (Segawa et al., 2020), however, the effect of early intervention of capsaicin in salt sensitive hypertension and its mechanism remain unexplored.

Thus, the present study was designed to investigate whether early intervention with capsaicin reduces salt sensitive hypertension and investigate Capsaicin pretreatment attenuates salt-sensitive hypertension by alleviating AMPK/Akt/Nrf2 pathway in the PVN.

## 2 Materials and methods

### 2.1 Ethical approval and animal

Eight-week-old male Dahl salt-sensitive (S) rats (Charles River Laboratories International, Inc., Wilmington, MA, United States) were used in this study. All procedures complied with the Guide for the Care and Use of Laboratory Animals (NIH publication, 8th edition, 2011). The animal study protocol was approved by the Animal Care and Use Committee of Xi’an Jiaotong University (protocol code No. 2020–62 and date of approval 25 March 2020) (Gao et al., 2023).

### 2.2 Experimental design

After 1 week of acclimatization, Dahl S rats were divided into four groups according to diet type: (1) NSD + PVN vehicle group (*n* = 7): rats were fed with food containing 0.3% NaCl and PVN infusion with vehicle; (2) NSD + capsaicin group (*n* = 7): rats were fed with food containing 0.3% NaCl and PVN infusion with capsaicin; (3) HSD + PVN vehicle group (*n* = 7): rats were fed with food containing 8% NaCl and PVN infusion with vehicle; (4) HSD + capsaicin group (*n* = 7): rats were fed with food containing 8% NaCl and PVN infusion with capsaicin. The determination of the effective dose of capsaicin is based on our pilot study combined with previous studies (Qi et al., 2022; Gao et al., 2023).

### 2.3 PVN microinjection

The osmotic minipumps (Qi et al., 2016) were implanted subcutaneously and connected to the PVN cannulae for the continuous infusion of capsaicin (purity ≥99%, Saint Louis, MO, United States) at 2 μg/h or vehicle for 6 weeks. PVN microinjections were performed bilaterally as described previously (Sun et al., 2016; Qi et al., 2022).

### 2.4 Blood pressure measurement

Before preparing to collect data, we will allow all rats to adapt to the experimental equipment for a week. Animals will soon become familiar with the program of this monitoring system and remain quiet in the monitoring equipment, greatly improving the accuracy of the data. The entire experimental process is standardized and timed by dedicated personnel to reduce experimental errors (Yu et al., 2021; Qi et al., 2022).

### 2.5 Collection of tissue samples

At the end of experiment, animals were sacrificed and the samples including brain tissue, blood, heart and arteries were collected, processed and stored for further analysis. The PVN samples were was isolated from rat brains in both sides of the PVN following Palkovits’s microdissection procedure as previously described (Palkovits, 1973; Su et al., 2022).

### 2.6 Hematoxylin and eosin (H&E) staining of heart and the renal structure

The HE staining is a staining method for tissue pathology, which can clearly distinguish the structure of tissues and cells through staining, in order to observe the morphology of tissues and cells under a microscope. This study used HE staining to observe the effects of long-term high salt diet on the structure of the heart and arteries in rats. The process of HE staining was performed as previously described (Liao et al., 2022).

### 2.7 Measurement of brain reactive oxygen species (ROS)

A red fluorescent reactive oxygen probe, dihydroethidium (DHE), was used to detect the ROS level in the PVN. The method for detecting ROS production in the PVN followed previous study using frozen unfixed brain sections (Qi et al., 2022).

### 2.8 Immunofluorescence staining of PVN

To visually evaluate the expression level of positive cells of the Nrf2, HO-1, iNOS and p-PIKKβ in the PVN, we used immunofluorescence staining to demonstrate the changes in and localize these indicators. Part of animals whose brains were assigned for immunochemistry assay was perfused transcardially with fresh normal saline followed by 4% paraformaldehyde. The brains were post fixed and treated with sucrose for cryoprotection. To perform immunofluorescence, fixed brain tissue sections containing the PVN were permeabilized with 0.3% Triton X-100 and blocked with 10% goat serum for 60 min at 37°C, then incubated with primary antibodies including the Nrf2 (ab62352, Abcam, Cambridge, MA, United States) and iNOS (ab49999, Abcam, Cambridge, MA, United States), HO-1 (ab52947, Abcam, Cambridge, MA, United States) and p-PIKKβ (sc-20781, Santa Cruz, TX, United States) overnight at 4°C. Next day, after washing of the primary antibody, the tissues were incubated with FITC-conjugated secondary antibody (ABC Staining System Kit, Santa Cruz) for 1 h at 37\u00B0C then covered for microscopy. NIH Image J software was used for cell counts and to quantify the fluorescence intensity of related molecular indicators in the PVN (Souza et al., 2019; Li et al., 2021).

### 2.9 Quantification of mRNA levels of pro-inflammatory cytokine

Messenger RNA (mRNA) expression levels in heart and PVN were assessed by real-time PCR (Li et al., 2021). The total RNA were isolated from the PVN and heart snap frozen tissues using Trizol (Invitrogen Inc.). After conversion of RNA to complementary cDNA, the mRNA level was quantified through quantitative RT-PCR. The expression atrial natriuretic peptide (ANP) and brain natriuretic peptide (BNP) was assessed from the left ventricle tissues and that of NOX2, NOX4, iNOS, tumor necrosis factor-α (TNF-α), Monocyte Chemoattractant Protein-1 (MCP-1), interleukin 6 (IL-6), interleukin 1β (IL-1β) were detected in PVN tissue. Primer sequences used for gene expression analysis are listed in Table 1.

**Table 1:** Rat primers used for real-time RT-PCR.

Rat genes	Forward (5′-3′)	Reverse (5′-3′)
ANP	CAGAAGCTGCTGGAGCTGATAAG	TGTAGGGCCTTGGTCCTTTG
BNP	GAAGGTGCTGTCCCAGATGA	CCAGCAGCTGCATCTTGAAT
NOX2	CTGCCAGTGTGTCGGAATCT	TGTGAATGGCCGTGTGAAGT
NOX4	GGATCACAGAAGGTCCCTAGC	AGAAGTTCAGGGCGTTCACC
iNOS	CCTTGTTCAGCTACGCCTTC	GGTAGCCCGAGTTCTTTCA
MCP-1	GTGCTGACCCCAATAAGGAA	TGAGGTGGTTGTGGAAAAGA
IL-1β	GCAATGGTCGGGACATAGTT	AGACCTGACTTGGCAGAGGA
IL-6	TCTCTCCGCAAGAGACTTCCA	ATACTGGTCTGTTGTGGGTGG
TNF α	ACCACGCTCTTCTGTCTACTG	CTTGGTGGTTTGCTACGAC
GAPDH	AGACAGCCGCATCTTCTTGT	CTTGCCGTGGGTAGAGTCAT

### 2.10 ELISA assay

The activity of NOX (ab186031, Abcam, Cambridge, United Kingdom) in the PVN were measured with the ELISA kits.

### 2.11 Western blotting

The total proteins were extracted from the punched PVN tissues and quantified. Total protein were separated on SDS-page gel through electrophoresis and transferred to the PVDF membranes, which were incubated with the following primary antibodies: total AMPK, and phosphorylated AMPK, total PI3K and phosphorylated PI3K (Abcam, Cambridge, United Kingdom), total Akt and phosphorylated Akt (Santa Cruz, TX, United States) (Cell Signaling Technology). Image J software was used for the quantification of western blotting images (Xu et al., 2020; Li et al., 2021).

### 2.12 Data analysis and statistics

GraphPad Prism 8.0 (GraphPad Software, La Jolla, CA, United States) was used to analyze the data. Analysis of blood pressure related data was performed using repeated-measures ANOVA. The other results were analyzed with two-way ANOVA with Turkey’s multiple comparison tests. Data were presented as means ± standard errors of the mean. Differences were considered significant at *p-values* < 0.05.

## 3 Results

### 3.1 Capsaicin pretreatment delayed blood pressure elevation and heart rate induced by high salt diet in Dahl S rats

**Figure 1:**
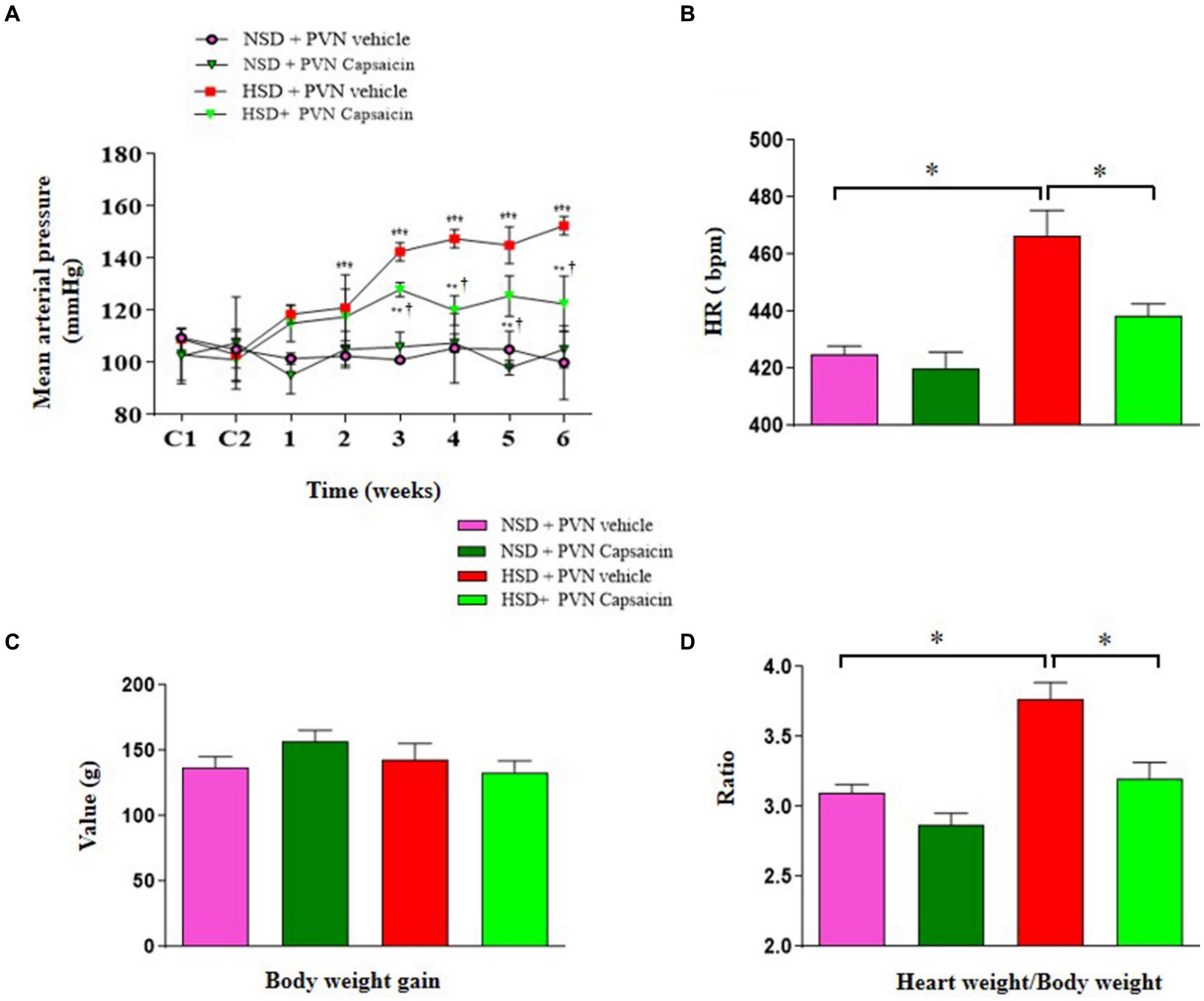
Capsaicin slowed down **(A)** MAP, **(B)** HR, **(C)** body weight gain and **(D)** heart weight/body weight of Dahl salt-sensitive (Dahl S) rats on 6 weeks of a normal-salt diet (NSD; 0.4% NaCl) or high-salt diet (HSD; 8% NaCl) diet. The data are expressed as the means ± S.E.M (7 per group). ^*^p < 0.05, ^**^p < 0.01, and ^***^p < 0.001 compared with group NSD; ^†^*p* < 0.05 compared with group HSD. NSD, normal salt diet; HSD, high salt diet.

Salt sensitivity is marked by variation in blood pressure parallel to salt consumption. In this experiment, we assessed change in blood pressure and heart rate following high salt intake. We found that high salt diet increased blood pressure as early as day 14 of treatment until the end of the experiment (Figure 1A), increased heart rate (Figure 1B) and Heart weight/Body weight (Figure 1D), but there was no change in weight gain (Figure 1C). Experimental and clinical evidence suggested that reducing salt intake can lower blood pressure in patients with hypertension even those with normal blood pressure (He and MacGregor, 2010; Kurtz et al., 2018). Interestingly, capsaicin pretreatment (beginning the third week) significantly attenuated blood pressure elevation and slowed the heart rate, reduced body weight gain in the HSD rats.

### 3.2 Capsaicin pretreatment alleviated vascular structure and cardiac hypertrophy induced by high salt diet in Dahl S rats

**Figure 2:**
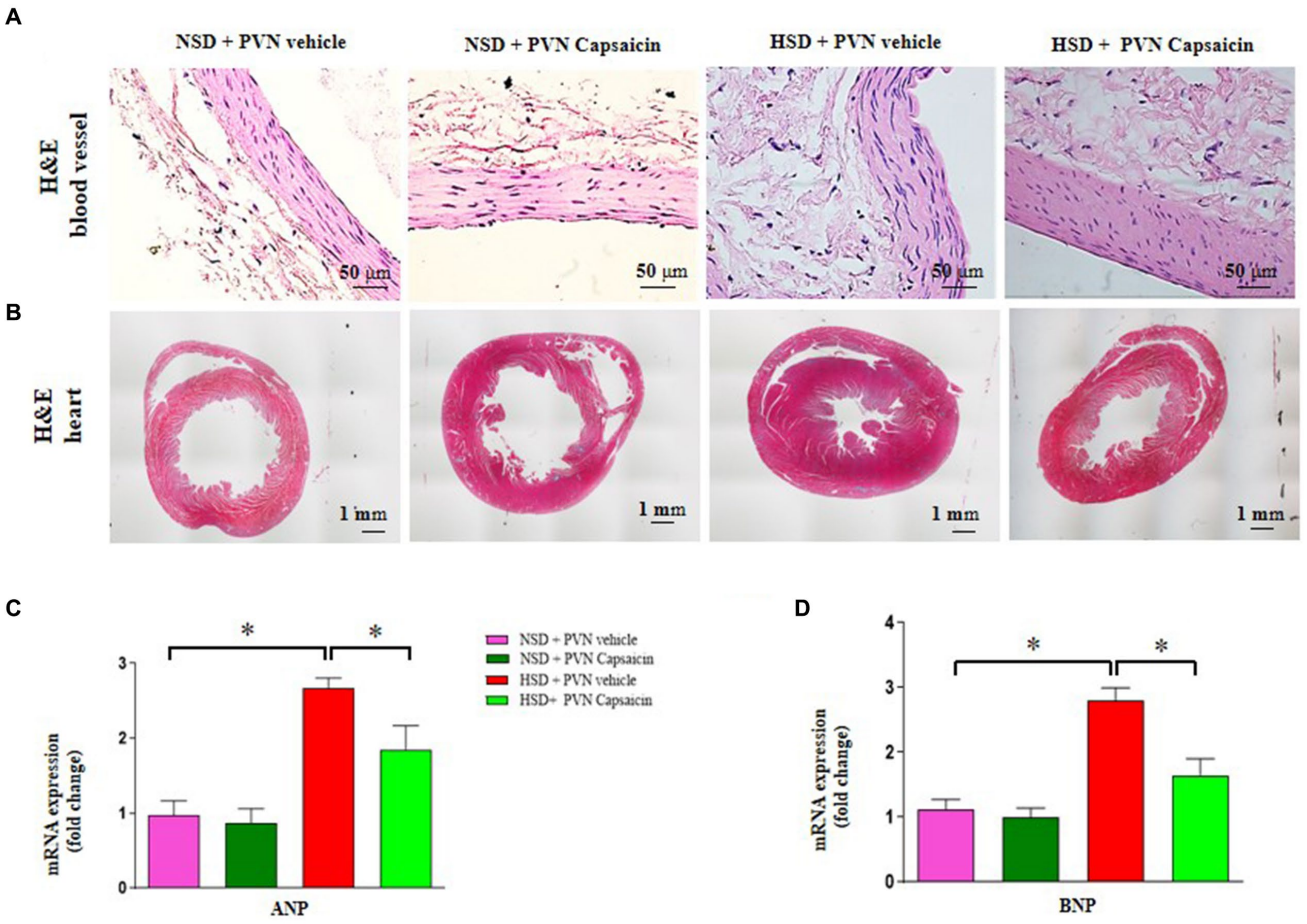
Capsaicin ameliorated **(A)** the thickness of vascular wall, **(B)** cardiac hypertrophy, **(C)** the mRNA levels of ANP **(D)** and BNP of Dahl salt-sensitive (Dahl S) rats on 6 weeks of a normal-salt diet (NSD; 0.4% NaCl) or high-salt diet (HSD; 8% NaCl) diet. The data are expressed as the means ± S.E.M (7 per group). ^*^p < 0.05, ^**^p < 0.01, and ^***^p < 0.001 compared with group NSD; NSD, normal salt diet; HSD, high salt diet.

Given that hypertension itself may cause cardiac hypertrophy and increase the risk of angina and myocardial infarction (Mavrogeni et al., 2022). This study was designed to observe the relationship of salt vascular and heart function. In this study, HE staining was used to show that the changes in vascular structure and cardiac hypertrophy. The elastic membrane in the middle layer of the aorta of the control group rats is arranged neatly and tightly; The total number of VSMCs in the middle layer of the aortic wall of the model group rats increased, and the arrangement of the elastic modulus in the middle layer was disordered and relaxed, The middle membrane of the capsaicin pretreatment rats slightly thickened, and the muscle layer cells were arranged neatly in the HSD rats (Figure 2A). Hematoxylin and eosin (H&E) staining showed increased thickness of ventricular walls and shrunken heart chambers of the rats in the HSD group compared with those of NSD rats (Figure 2B). In addition, the gene levels of ANP and BNP the gene expression levels were higher in rats of HSD group compared with those of NSD rats (Figures 2C,D). Capsaicin pretreatment significantly reversed vascular and cardiac hypertrophy in the HSD rats.

### 3.3 Capsaicin pretreatment enhanced anti-oxidative capacity in the PVN in Dahl S rats

**Figure 3:**
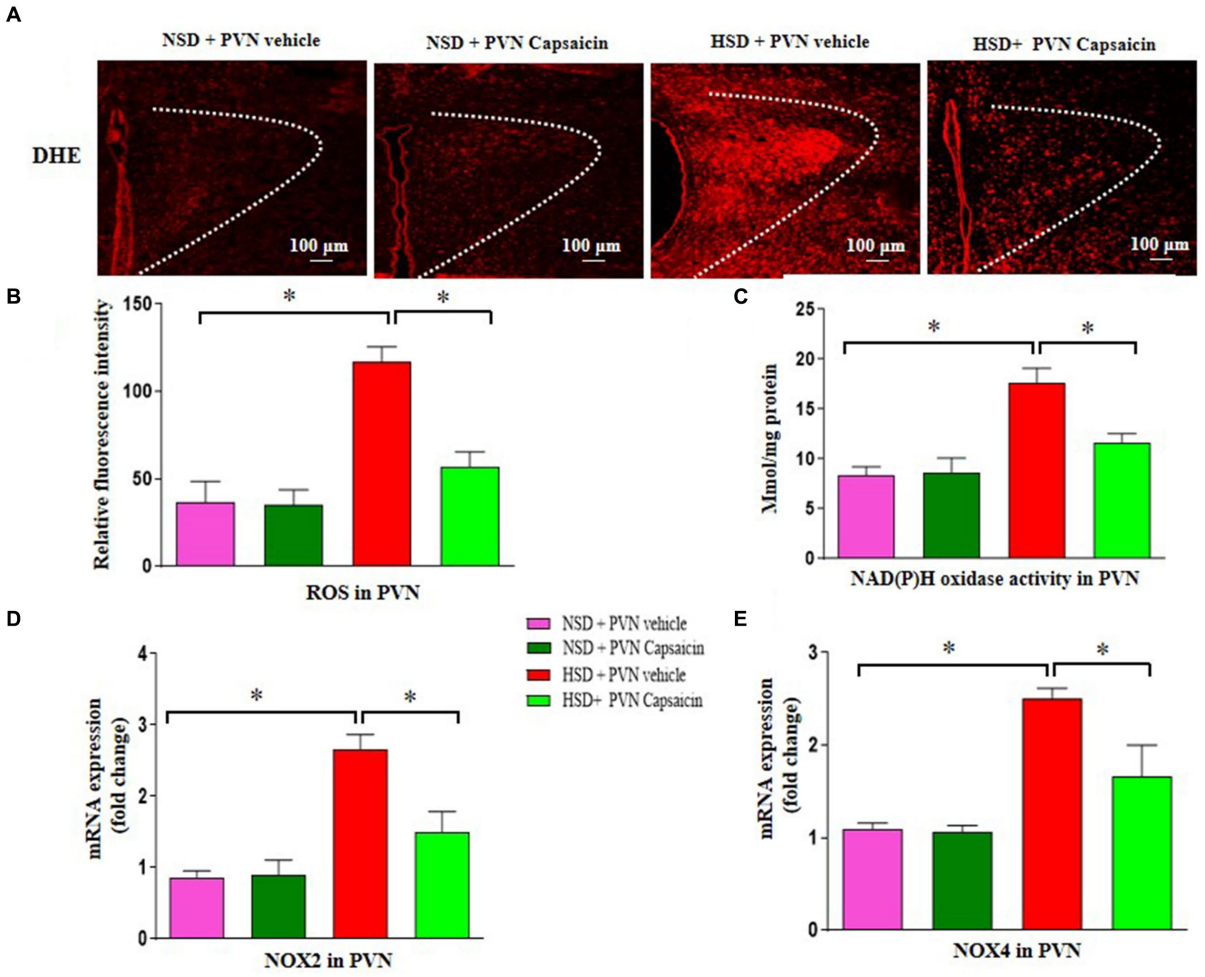
Capsaicin decreased **(A,B)** the level of reactive oxygen species, **(C)** NAD(P)H oxidase activity, **(D)** the mRNA levels of NOX2 **(E)** and NOX4 in the PVN of Dahl salt-sensitive (Dahl S) rats on 6 weeks of a normal-salt diet (NSD; 0.4% NaCl) or high-salt diet (HSD; 8% NaCl) diet. The data are expressed as the means ± S.E.M (7 per group). ^*^p < 0.05, ^**^p < 0.01, and ^***^p < 0.001 compared with group NSD; NSD, normal salt diet; HSD, high salt diet.

Research has found that consuming capsaicin for 4 weeks can enhance the antioxidant capacity of serum lipoproteins in adult men and women, and the antioxidant properties of capsaicin have additional advantages in the treatment of cardiovascular diseases (Pasierski and Szulczyk, 2022). Our results showed that ROS production (Figures 3A,B), the NAD (P) H oxidase activity (Figure 3C), NOX2 gene expression (Figure 3D) and t NOX4 gene expression (Figure 3E) were increased compared with those of NSD rats. Capsaicin pretreatment significantly reversed the changes in the above indicators in the HSD rats.

### 3.4 Capsaicin pretreatment alleviated high salt-inhibited the positive cells of Nrf2 and HO-1 expression in the PVN in Dahl S rats

**Figure 4:**
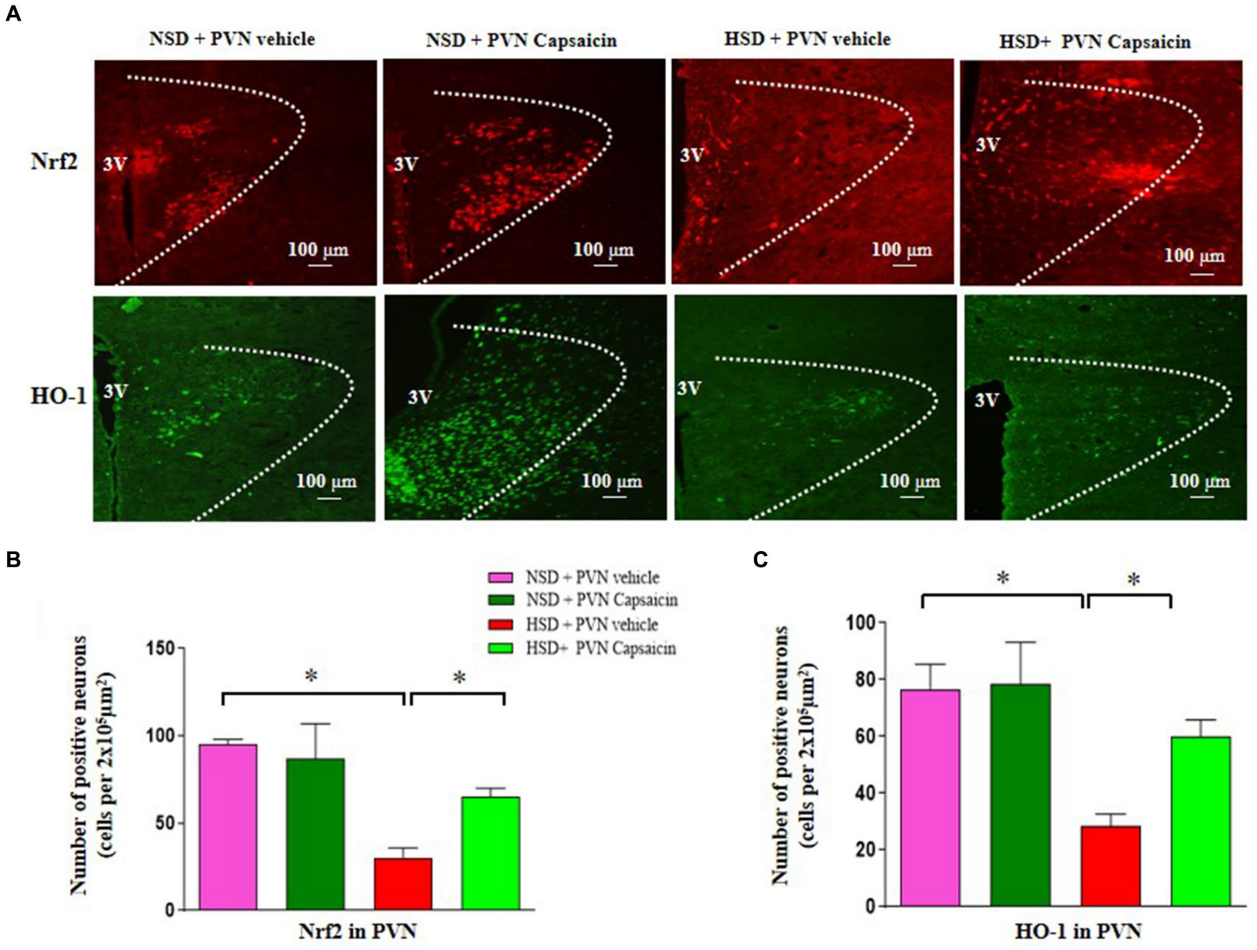
Capsaicin decreased **(A,B)** the number of Nrf2 positive cells in the PVN, **(A,C)** the number of HO-1 positive cells in the PVN in the PVN of Dahl salt-sensitive (Dahl S) rats on 6 weeks of a normal-salt diet (NSD; 0.4% NaCl) or high-salt diet (HSD; 8% NaCl) diet. The data are expressed as the means ± S.E.M (7 per group). ^*^p < 0.05, ^**^p < 0.01, and ^***^p < 0.001 compared with group NSD; NSD, normal salt diet; HSD, high salt diet.

Nrf2 is the most important known oxidative stress pathway that plays an important role when activated, reducing cell apoptosis, participating in neuroprotection, delaying aging, and reducing organ damage. Accumulating evidence revealed that Nrf2 pathway participated in the regulation of blood pressure and cardiovascular function. Our immunofluorescence studies revealed salt diet decreased number of Nrf2 and HO-1 positive cells compared with those of NSD rats (Figures 4A–C). Interestingly, capsaicin pretreatment significantly increased the number of the positive cells of Nrf2 and HO-1 in the PVN of HSD rats.

### 3.5 Capsaicin pretreatment attenuated NF-κB activity and positive cells of p-IKKβ expression in the PVN in Dahl S rats

**Figure 5:**
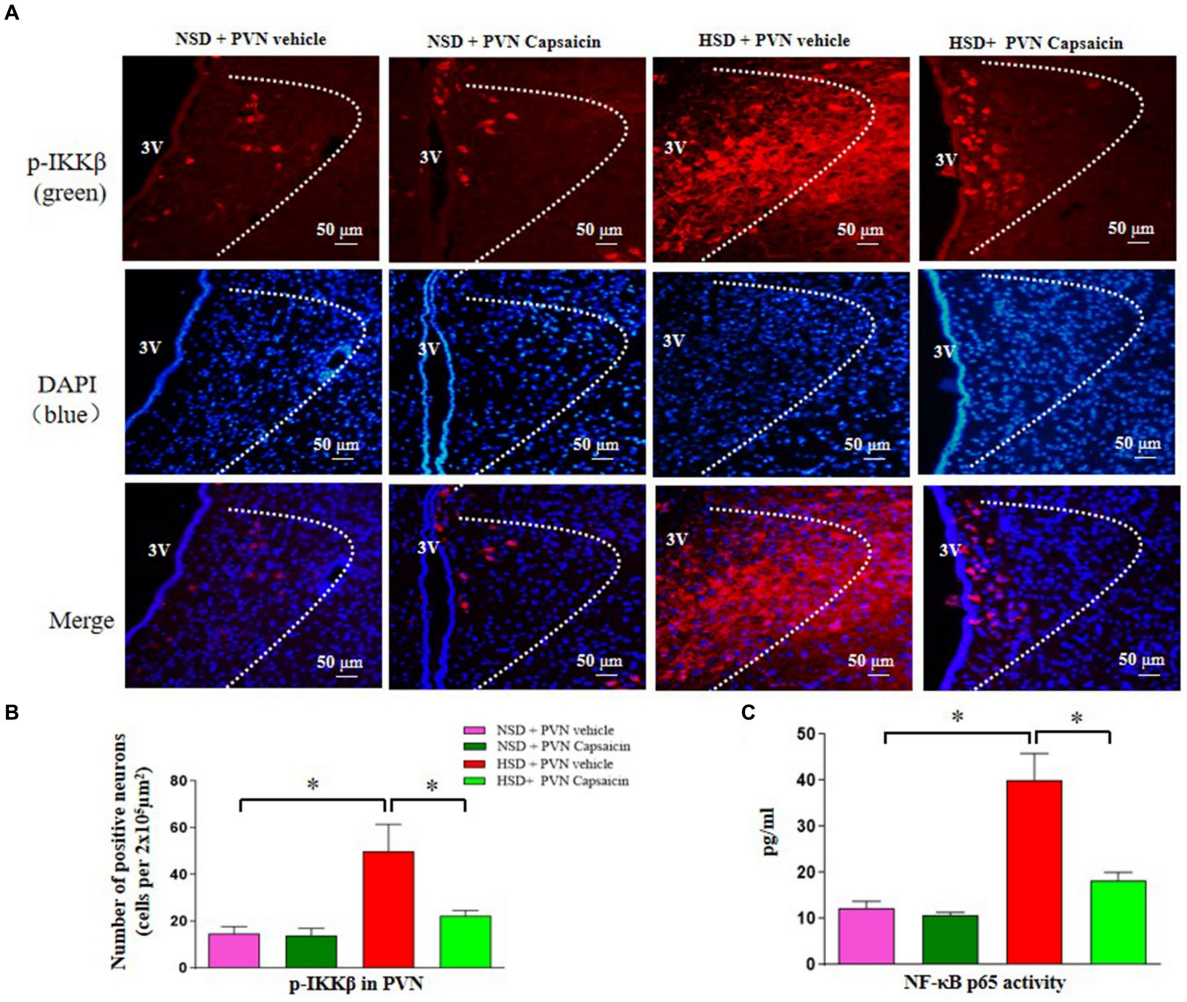
Capsaicin decreased **(A,B)** the number of p-IKKβ positive cells in the PVN, **(C)** NF-κB p65 activity in the PVN of Dahl salt-sensitive (Dahl S) rats on 6 weeks of a normal-salt diet (NSD; 0.4% NaCl) or high-salt diet (HSD; 8% NaCl) diet. The data are expressed as the means ± S.E.M (7 per group). ^*^p < 0.05, ^**^p < 0.01, and ^***^p < 0.001 compared with group NSD; NSD, normal salt diet; HSD, high salt diet.

The critical role of NF-κB activity in the PVN has been shown in cardiovascular diseases (Wang et al., 2019). In the present study, using the method of immunofluorescence staining and ELISA, we examined p-IKKβ positive cells and NF-κB p65 activity in the PVN. The results showed that the number of p-IKKβ positive cells (Figures 5A,B) and NF-κB p65 activity (Figure 5C) were increased in HSD rats compared with those of NSD rats. Interestingly, capsaicin pretreatment significantly decreased the number of p-IKKβ positive cells and NF-κB p65 activity in the PVN of HSD rats.

### 3.6 Capsaicin pretreatment reduced the expression of iNOS in the PVN in Dahl S rats

**Figure 6:**
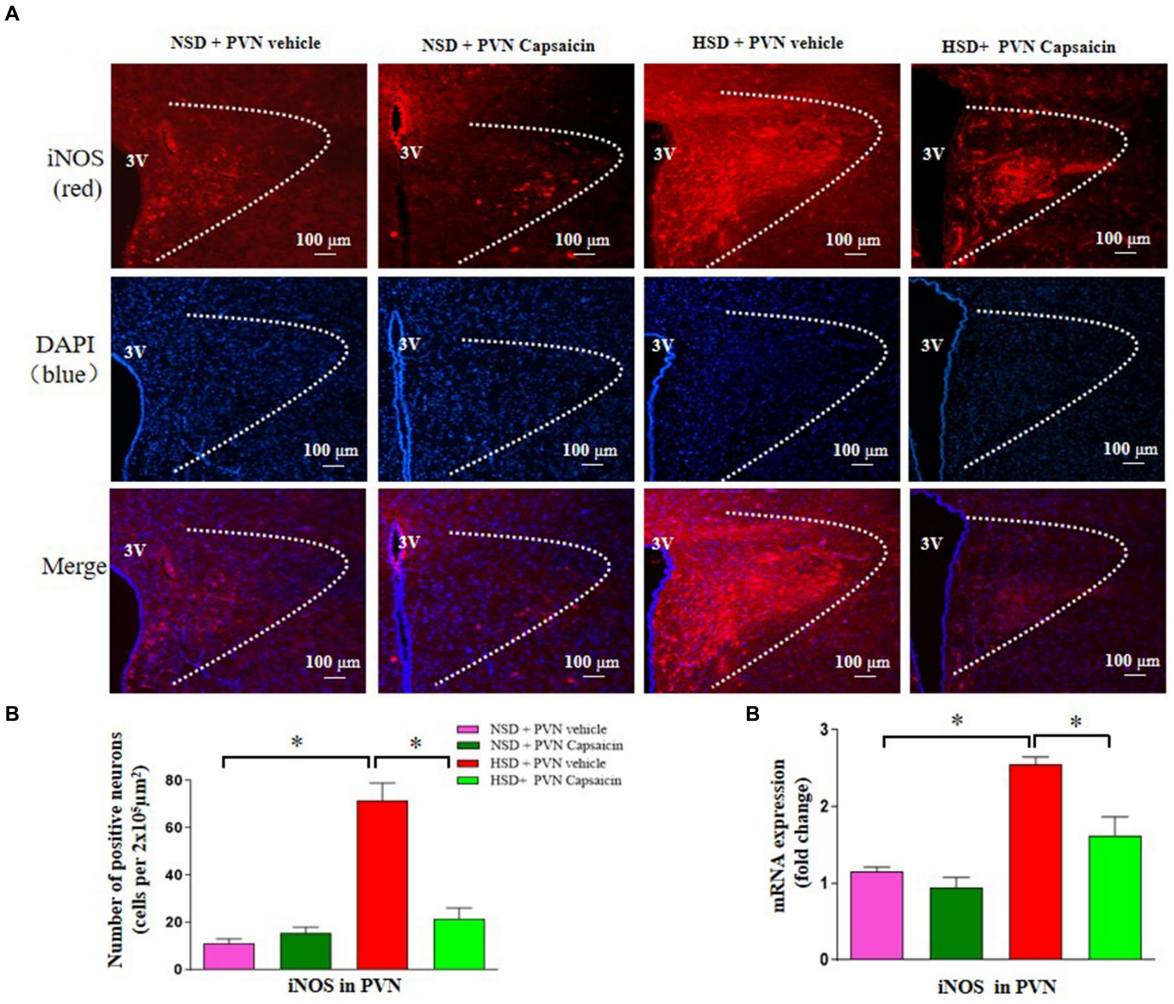
Capsaicin decreased **(A,B)** the number of iNOS positive cells in the PVN, **(C)** the mRNA levels of iNOS in the PVN of Dahl salt-sensitive (Dahl S) rats on 6 weeks of a normal-salt diet (NSD; 0.4% NaCl) or high-salt diet (HSD; 8% NaCl) diet. The data are expressed as the means ± S.E.M (7 per group). ^*^p < 0.05, ^**^p < 0.01, and ^***^p < 0.001 compared with group NSD; NSD, normal salt diet; HSD, high salt diet.

Currently, how to improve target organ damage caused by hypertension has attracted increasing attention from researchers, and the nitric oxide synthase nitric oxide system has become a hot research topic in recent years. Therefore, this study also used immunofluorescence and PCR technology to detect the expression of iNOS positive cells. The results showed that the number of iNOS positive cells (Figures 6A,B) and mRNA of iNOS (Figure 6C) were increased in the HSD rats. Interestingly, capsaicin pretreatment significantly decreased the number of iNOS the positive cells and the iNOS mRNA expression level in the PVN of HSD rats.

### 3.7 Capsaicin pretreatment decreased cytokines production in the PVN in Dahl S rats

**Figure 7:**
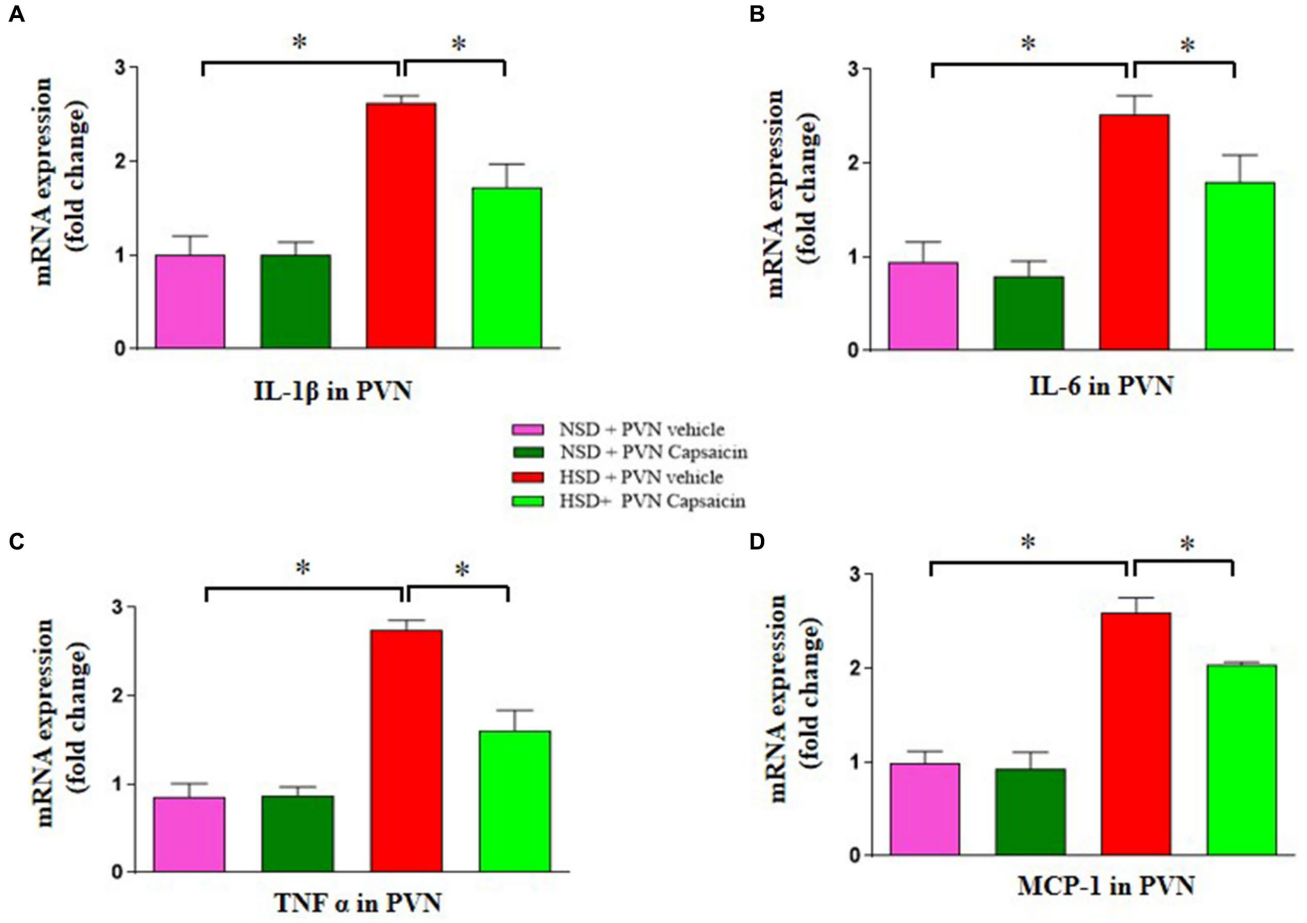
Capsaicin decreased **(A)** the mRNA levels of IL-1β, **(B)** the mRNA levels of IL-6, **(C)** the mRNA levels of TNF α and **(D)** the mRNA levels of MCP-1in the PVN of Dahl salt-sensitive (Dahl S) rats on 6 weeks of a normal-salt diet (NSD; 0.4% NaCl) or high-salt diet (HSD; 8% NaCl) diet. The data are expressed as the means ± S.E.M (7 per group). ^*^p < 0.05, ^**^p < 0.01, and ^***^p < 0.001 compared with group NSD; NSD, normal salt diet; HSD, high salt diet.

Capsaicin has shown cholesterol lowering properties in animal experiments. It is generally believed that High salt can lead to an increase in inflammatory cytokines in the PVN, thereby accelerating the progression of hypertension. Our results showed that the gene expression levels for TNFα (Figure 7A), IL-1β (Figure 7B), MCP-1(Figure 7C), and IL-6 (Figure 7D) were elevated in the PVN of HSD group. Capsaicin pretreatment significantly reversed the above pro-inflammatory cytokine indicators in the PVN of in the HSD rats.

### 3.8 Capsaicin pretreatment increased AMPK phosphorylation and attenuated p-PI3K and p-AKT expression in the PVN in Dahl S rats

**Figure 8:**
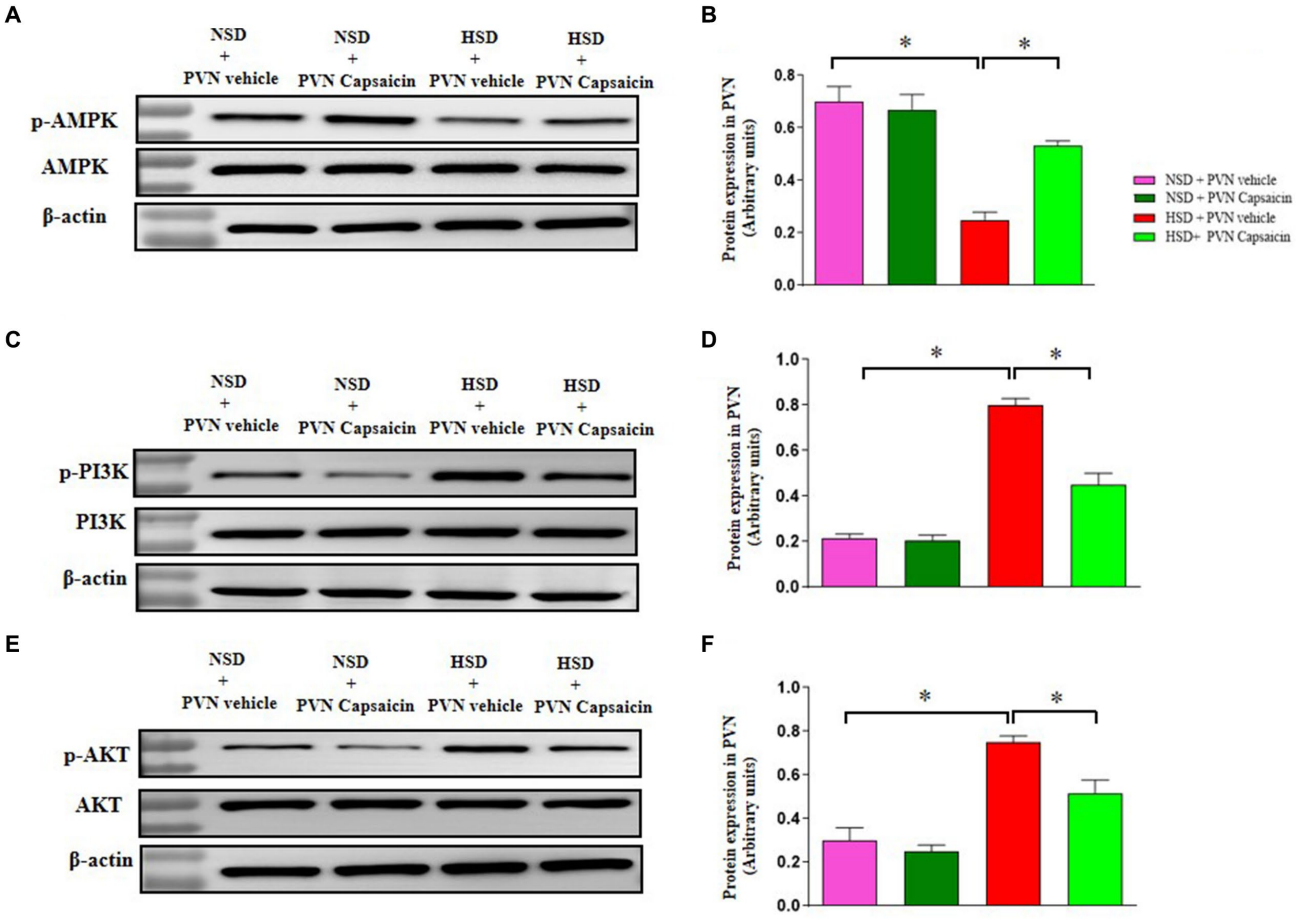
Capsaicin ameliorated **(A,B)** the protein expression of p-AMPK, **(C,D)** the protein expression of p-PI3K, **(E,F)** the protein expression of p-AKT in the PVN of Dahl salt-sensitive (Dahl S) rats on 6 weeks of a normal-salt diet (NSD; 0.4% NaCl) or high-salt diet (HSD; 8% NaCl) diet. The data are expressed as the means ± S.E.M (7 per group). ^*^p < 0.05, ^**^p < 0.01, and ^***^p < 0.001 compared with group NSD; NSD, normal salt diet; HSD, high salt diet.

PI3K/Akt signaling pathway can regulate the activity of Nrf2. Akt phosphorylation further helps Nrf2 achieve nuclear plasma shuttle, thereby enhancing endogenous antioxidant activity in cells (Wang et al., 2019; Bianchi et al., 2023). Our results showed protein expression of high salt diet inhibited the phosphorylation of AMPK (Figures 8A,B) and while enhancing the phosphorylation of PI3K (Figures 8C,D) and AKT (Figures 8E,F) in PVN of HSD group. Capsaicin pretreatment significantly promoted the AMPK phosphorylation and attenuated that of PI3K and AKT in the PVN of HSD rats.

## 4 Discussion

Capsaicin and related compounds form a naturally occurring chemical group called capsaicin, which give chili peppers a unique spicy taste, and are believed to constitute a natural defense of plants against herbivores and fungi (Cheng et al., 2023). Interest to study their role in cardiovascular diseases is increasing among scholars (Gualtieri et al., 2023; Zaleski et al., 2023). On the other hand, the role the PVN, in the occurrence, development, and prognosis of hypertension is largely acknowledged (Souza et al., 2019; Li et al., 2021; Gao et al., 2023), however, whether capsaicin influence the course of salt-induced hypertension through the PVN remains unclear. Designed to dissipate this concern, the present study yielded two novel findings: (i) Capsaicin can inhibit the progression of blood pressure, and improve vascular structure and cardiac dysfunction in salt-induced hypertensive rats. (ii) Capsaicin pretreatment attenuates salt-sensitive hypertension by alleviating AMPK/Akt/Nrf pathway in the PVN.

To date, although hypertension is a risk factor for cardiovascular disease death, it is also a preventable and controllable factor for cardiovascular disease events (Prenissl et al., 2019). Although the pathogenesis of hypertension is not yet clear, it has been recognized that hypertension is closely related to lifestyle and dietary habits (Yang et al., 2023). Accumulating evidence supports that consumption of too much salt constitutes one of the risk factors of hypertension (Vargas-Meza et al., 2023). Among people with high salt diet habits, the incidence of hypertension is high, which supports the positive correlation between high sodium salts intake and hypertension and makes high salt an important pathogenic factor for hypertension. For the prevention of hypertension or attenuation of hypertension in patients, a low salt diet is recommended, which means a daily salt intake of <6 g. Randomized trials have shown that reducing salt intake can not only improve hypertension in patients, but also lowered blood pressure in patients with normal blood pressure (He and MacGregor, 2010; He et al., 2020). High salt levels activate complex pathological mechanisms involved in numerous diseases. However, there is no clear delineation of such mechanisms. Consistent with previous reports, our data also showed that a long-term high salt diet can lead to a sustained increase in blood pressure, damage cardiac function in rats.

AMPK is an AMP dependent protein kinase which exists in every cell and is present in all organisms, from yeast to humans. At the same time, AMPKis an energy sensor that can detect energy levels within cells and respond accordingly. AMPK is synthesized in many organ cells, including the liver, brain, adipocytes, and muscle cells. Activating AMPK can be used to improve many conditions, including chronic inflammation, diabetes, obesity, cardiovascular disease and mitochondrial disease (Han et al., 2021; Guo et al., 2022). In different animal models, the activation and inhibition of AMPK were closely related to oxidative stress and inflammatory responses (Yu et al., 2020). A couple of studies have reported that reducing inflammation can also activate AMPK. Inflammatory cytokines can inhibit AMPK, while anti-inflammatory cytokines can activate AMPK (Huang et al., 2021). In this work, we found that high salt was associated with increased inflammatory markers including mRNA of TNF and IL1β, NF-κB and reduced AMPK phosphorylation in the PVN. However, capsaicin could revert such molecular changes suggesting a clear relationship between capsaicin and AMPK as reported previously by many experimental data in prostate cells and aging endothelial cell (Saha et al., 2022). Capsaicin ameliorates phospho-AMPK among numerous animal models such as endothelial cell aging (Zhu et al., 2022). Consistent with previous reports, this study showed the novel central mechanism of capsaicin. We found that capsaicin pretreatment obviously upregulated p-AMPK/AMPK expression were evaluated by western blotting in the PVN of the hypertensive rats.

The PI3K/Akt signaling pathway is one of the most important intracellular signaling pathways in mammals, playing an important role in regulating various cellular functions, including metabolism, inflammation, oxidative stress, growth, and protein synthesis, but associated with diseases such as hypertension. Thus, the expression of key proteins in the PI3K/Akt pathway in spontaneously hypertensive rat PVN was significantly increased (Shi et al., 2023), which promotes hypertension via sympathetic activation and vasopressin release. In the same hypertensive animal model, relaxin was shown to promote sympathetic nerve activation and blood pressure elevation from the PVN by stimulating PI3K expression and enhancing Akt phosphorylation However, PI3K inhibitor, LY294002, or Akt inhibitor (MK-2206) can eliminate the effects of relaxin on renal sympathetic nervous activity, mean arterial pressure, and plasma norepinephrine (Bian et al., 2021). This effect of PI3K/Akt in the PVN is not limited to hypertension. Thus, other studies have shown that the phosphorylation of PI3K and Akt proteins in PVN of myocardial infarction rats was significantly increased, and the application of LY294002 can inhibit the phosphorylation of PI3K and Akt, while also inhibiting peripheral sympathetic nerve activity in rats (Gao et al., 2023). Findings from the above studies support our data which have showed that high salt diet increased p-PI3K and p-Akt/Akt protein expression in the rats and that capsaicin pretreatment could attenuate such phosphorylation in the PVN thereby attenuating blood pressure raise in Dahl S Rats.

Change in the Nrf2 expression and activation in the brain has been associated with the hypertension while targeting it offers therapeutic effect. Our data showed that high salt-induced hypertension was associated with reduced Nrf2 level in the PVN, which was improved by capsaicin. Our finding agrees with number of previous reports. A previous study has shown that reduction in nuclear translocation of Nrf2 in the rostral ventrolateral medulla (RVLM) neurons contributes to hypertension induced by LPS-mediated systemic inflammation (Wu et al., 2016). Experiments have confirmed that Nrf2 activation can improve oxidative stress imbalance and reduce ROS levels (Li et al., 2023). Additionally, PVN administration of Tert-butylhydroquinone (tBHQ), a selective Nrf2 activator, could attenuate hypertension by targeting the Nrf2-mediated signaling pathway. Such effects were abrogated by knocking down Nrf2 expression in the PVN (Bai et al., 2017). Moreover, the anti-hypertensive effect of endogenous H_2_S in the PVN in SHR hypertension could be abolished by Nrf2 knockdown in the PVN (Xia et al., 2023). PI3K/Akt is an important member of the growth factor receptor superfamily signaling pathway that is an upstream signaling molecule of Nrf2, which can activate Nrf2 and regulate its downstream gene transcription. For example, PI3K activators can activate and promote Nrf2 nuclear translocation (Xie et al., 2023). Also, some neuroprotective agents were reported to activate Nrf2 by activating the PI3K/Akt signaling pathway in order to exert a neuroprotective effect on Parkinson’s model cells (Han et al., 2022). Exogenous stimuli can promote PI3K/Akt phosphorylation by activating tyrosine kinase receptors, thereby activating Nrf2 (Xu et al., 2022). There is experimental evidence that some neuroprotective agents can activate NrQ by activating the PI3K/Akt signaling pathway, exerting a neuroprotective effect on Parkinson’s model cells. It can be seen that the activation of Nrf2 is closely related to the apoptosis of PD cells mediated by the PI3K/Akt signaling pathway. One notable feature is the study of female animals, where low doses of capsaicin can protect follicles from apoptosis and atresia, and stimulate follicular development. Therefore, in female animals, low-dose capsaicin application can alleviate the systemic oxidation process during ovulation. It is well-known that increased oxidative stress in the PVN is involved in hypertension. In line with these findings, this study showed that capsaicin effectively exerts antioxidant effects in the central nervous system by reducing ROS production and the expression of NOX2 and NOX4 potentially through Nrf2 activation. This is evidenced by our result related to Nrf2 and HO-1 level in the PVN in response to capsaicin the intervention.

Collectively, capsaicin pretreatment alleviated salt-sensitive hypertension by alleviating AMPK/Akt/Nrf2 pathway, which inhibits ROS production, oxidative stress, Nrf2 and HO-1 and proinflammatory cytokines expression level in the PVN and subsequently lowered high salt intake-induced hypertension, arterial damage and cardiac dysfunction caused by hypertension.

## 5 Conclusion

**Figure 9:**
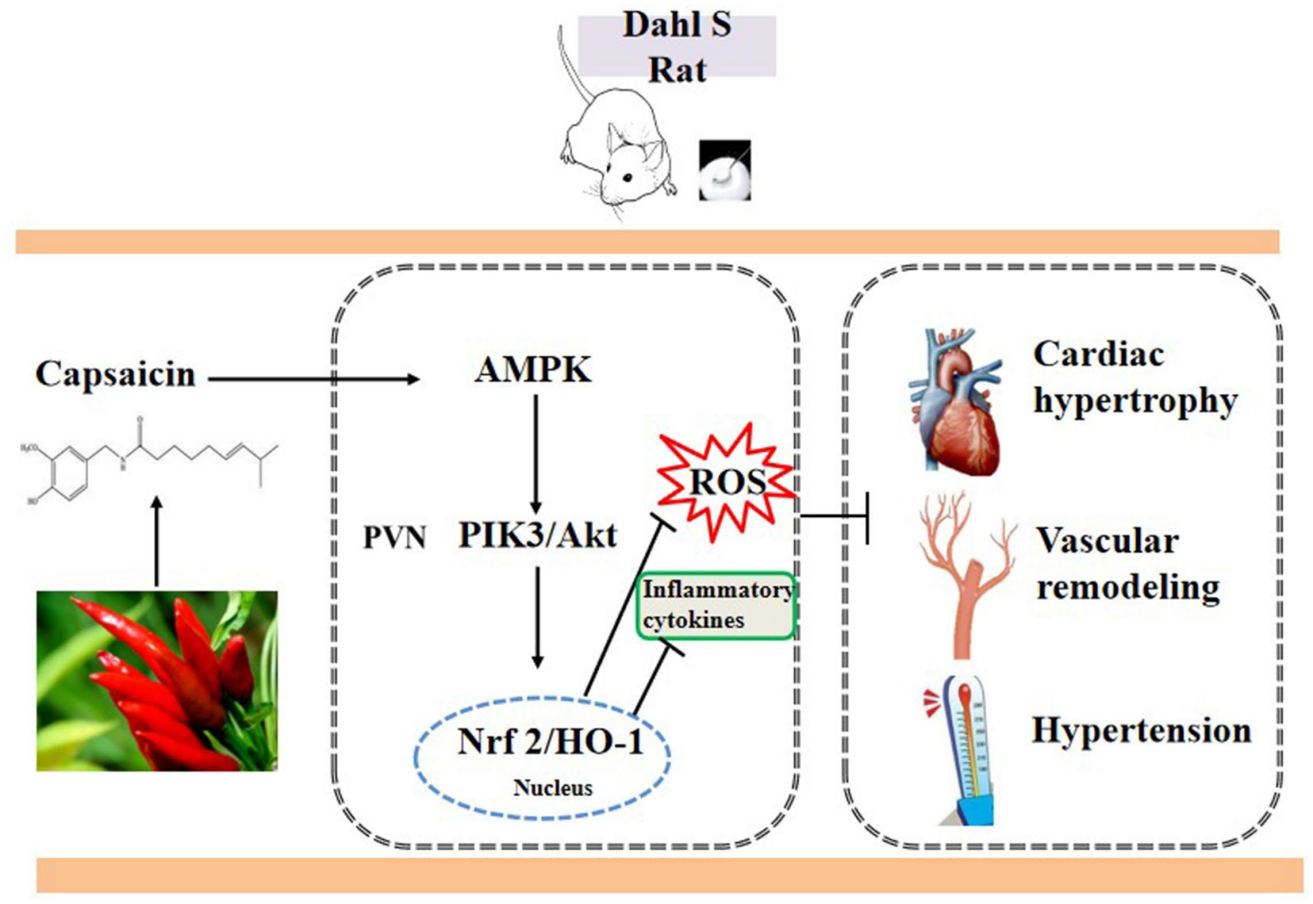
The schematic showing the mechanism of capsaicin pretreatment attenuates salt-sensitive hypertension by alleviating AMPK/Akt/Nrf2 pathway in hypothalamic paraventricular nucleus.

The AMPK/Akt/iNOS pathway in the PVN is involved in blood pressure and heart rate elevation. Our findings, for the first time, demonstrated that the effects of capsaicin were mediated by the AMPK/Akt/Nrf2 pathway-attenuated pro-inflammatory cytokine production and oxidative stress factors (Figure 9).

## Data availability statement

The original contributions presented in the study are included in the article/Supplementary material, further inquiries can be directed to the corresponding author.

## Ethics statement

The animal study was approved by Animal Care and Use Committee of Xi’an Jiaotong University (protocol code No. 2020–62 and date of approval 25 March 2020). The study was conducted in accordance with the local legislation and institutional requirements.

## Author contributions

X-YJ: Conceptualization, Data curation, Formal analysis, Investigation, Methodology, Project administration, Software, Writing – original draft, Writing – review & editing, Funding acquisition. YY: Methodology, Writing – review & editing. X-TJ: Conceptualization, Funding acquisition, Investigation, Methodology, Writing – original draft. D-LJ: Conceptualization, Data curation, Formal analysis, Resources, Writing – original draft. L-YF: Conceptualization, Formal analysis, Investigation, Methodology, Writing – review & editing. HT: Data curation, Project administration, Writing – review & editing. X-YY: Methodology, Writing – review & editing. X-YZ: Data curation, Software, Writing – review & editing. K-LL: Investigation, Methodology, Writing – review & editing. Y-MK: Funding acquisition, Supervision, Writing – review & editing. X-JY: Funding acquisition, Investigation, Supervision, Visualization, Writing – review & editing.
