# Supplementary material for: Capsaicin pretreatment attenuates salt-sensitive hypertension by alleviating AMPK/Akt/Nrf2 pathway in hypothalamic paraventricular nucleus
Source: Front Neurosci. 2024 May 30;18:1416522. doi: 10.3389/fnins.2024.1416522 (PMC11169651; doi:10.3389/fnins.2024.1416522)
Supplement: Supplementary file 1 [file Presentation_1.PPTX]

## Slide 1
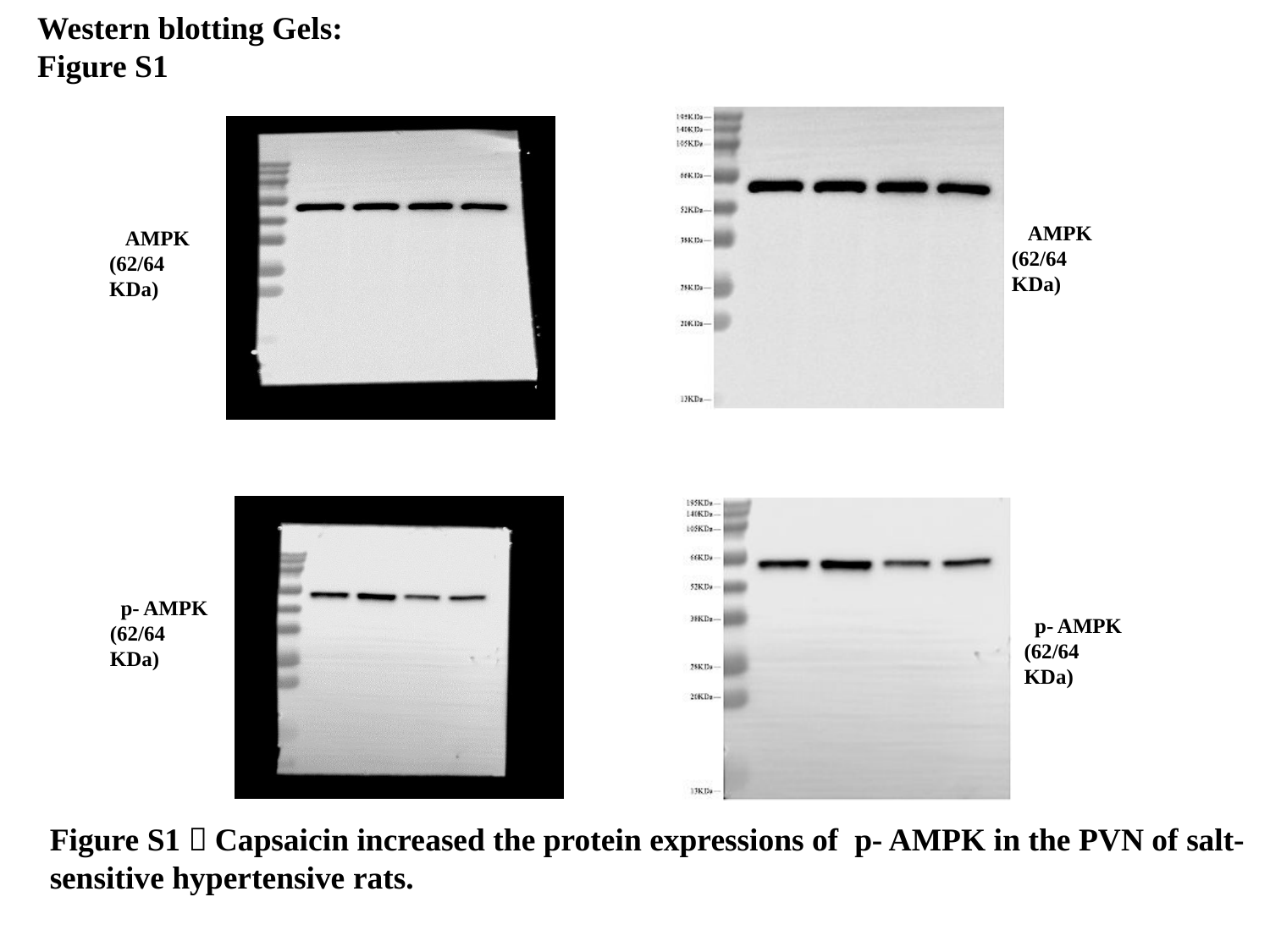

Western blotting Gels:
Figure S1
 AMPK
(62/64 KDa)
 AMPK
(62/64 KDa)
 p- AMPK
(62/64 KDa)
 p- AMPK
(62/64 KDa)
Figure S1：Capsaicin increased the protein expressions of p- AMPK in the PVN of salt-sensitive hypertensive rats.

## Slide 2
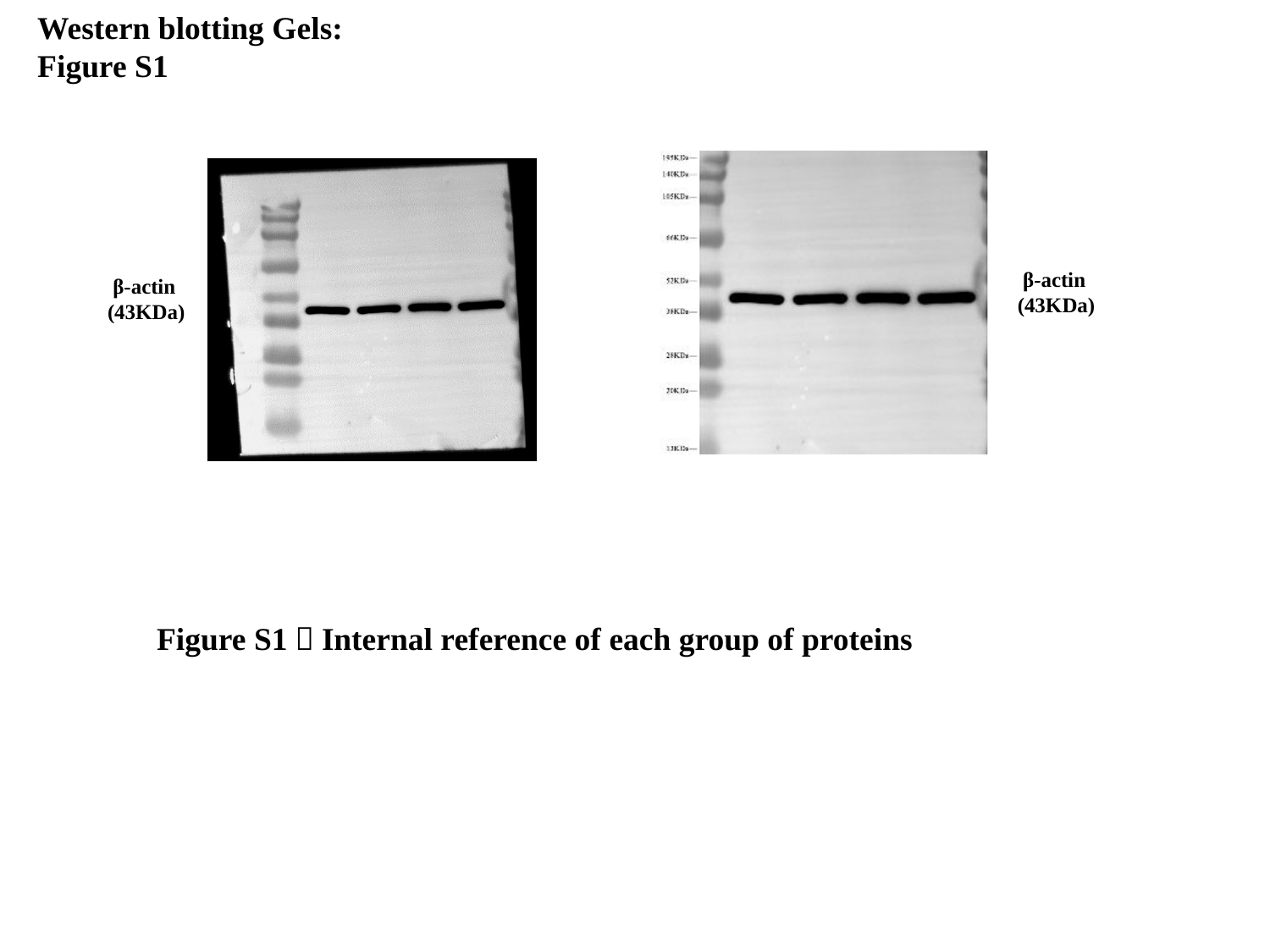

Western blotting Gels:
Figure S1
 β-actin
(43KDa)
 β-actin
(43KDa)
Figure S1：Internal reference of each group of proteins

## Slide 3
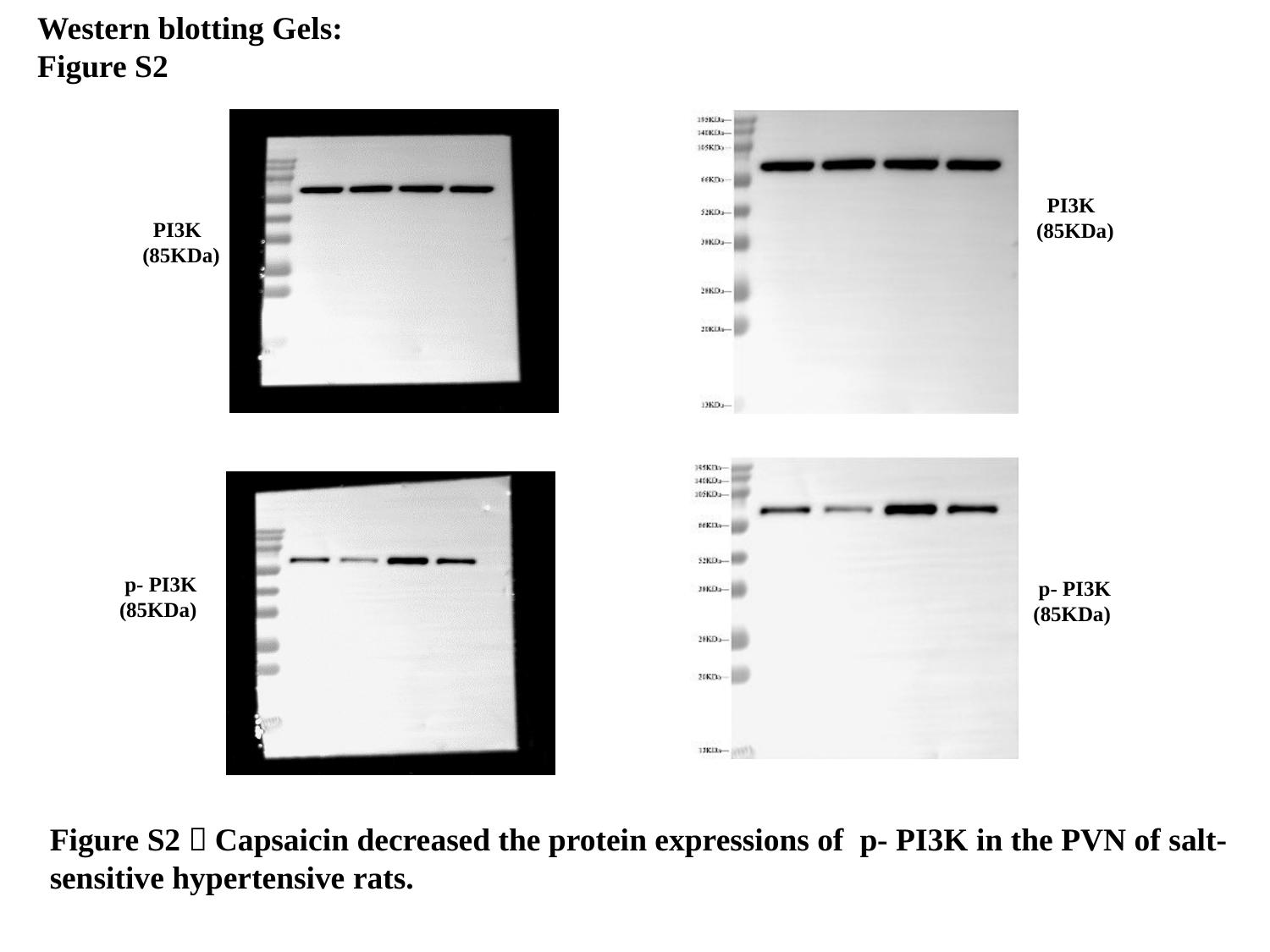

Western blotting Gels:
Figure S2
 PI3K
(85KDa)
 PI3K
(85KDa)
 p- PI3K
(85KDa)
 p- PI3K
(85KDa)
Figure S2：Capsaicin decreased the protein expressions of p- PI3K in the PVN of salt-sensitive hypertensive rats.

## Slide 4
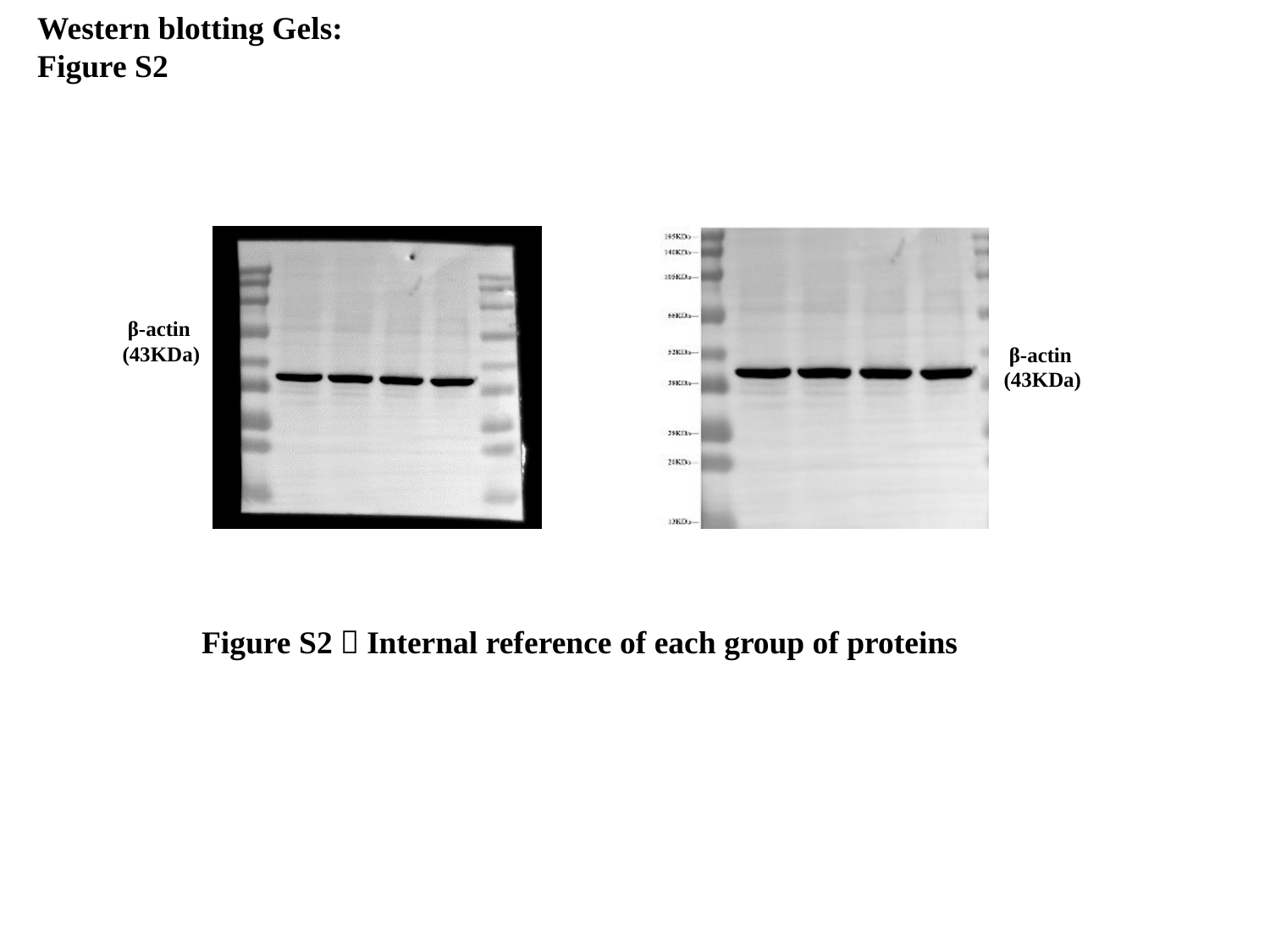

Western blotting Gels:
Figure S2
 β-actin
(43KDa)
 β-actin
(43KDa)
Figure S2：Internal reference of each group of proteins

## Slide 5
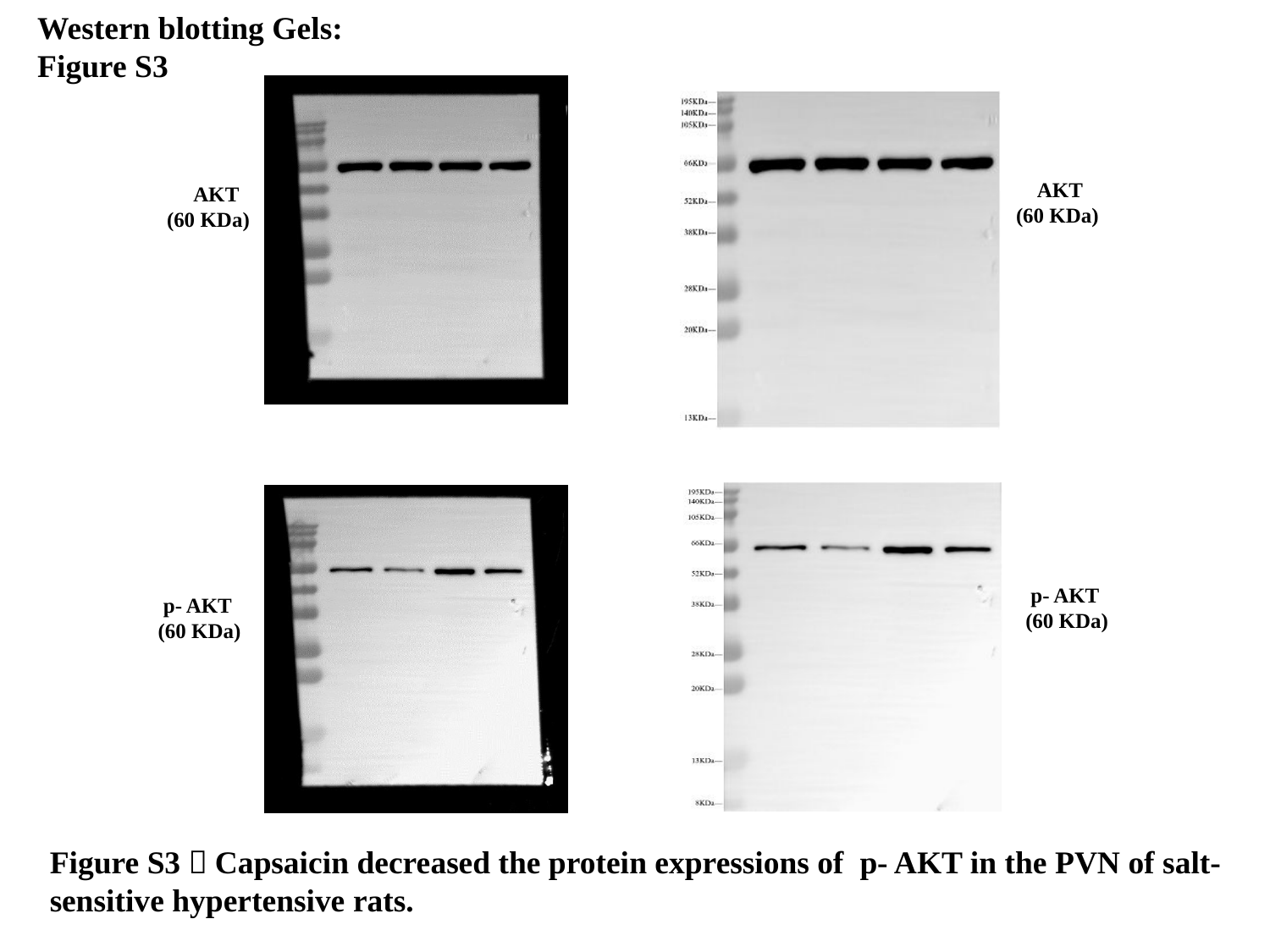

Western blotting Gels:
Figure S3
 AKT
(60 KDa)
 AKT
(60 KDa)
 p- AKT
 (60 KDa)
 p- AKT
 (60 KDa)
Figure S3：Capsaicin decreased the protein expressions of p- AKT in the PVN of salt-sensitive hypertensive rats.

## Slide 6
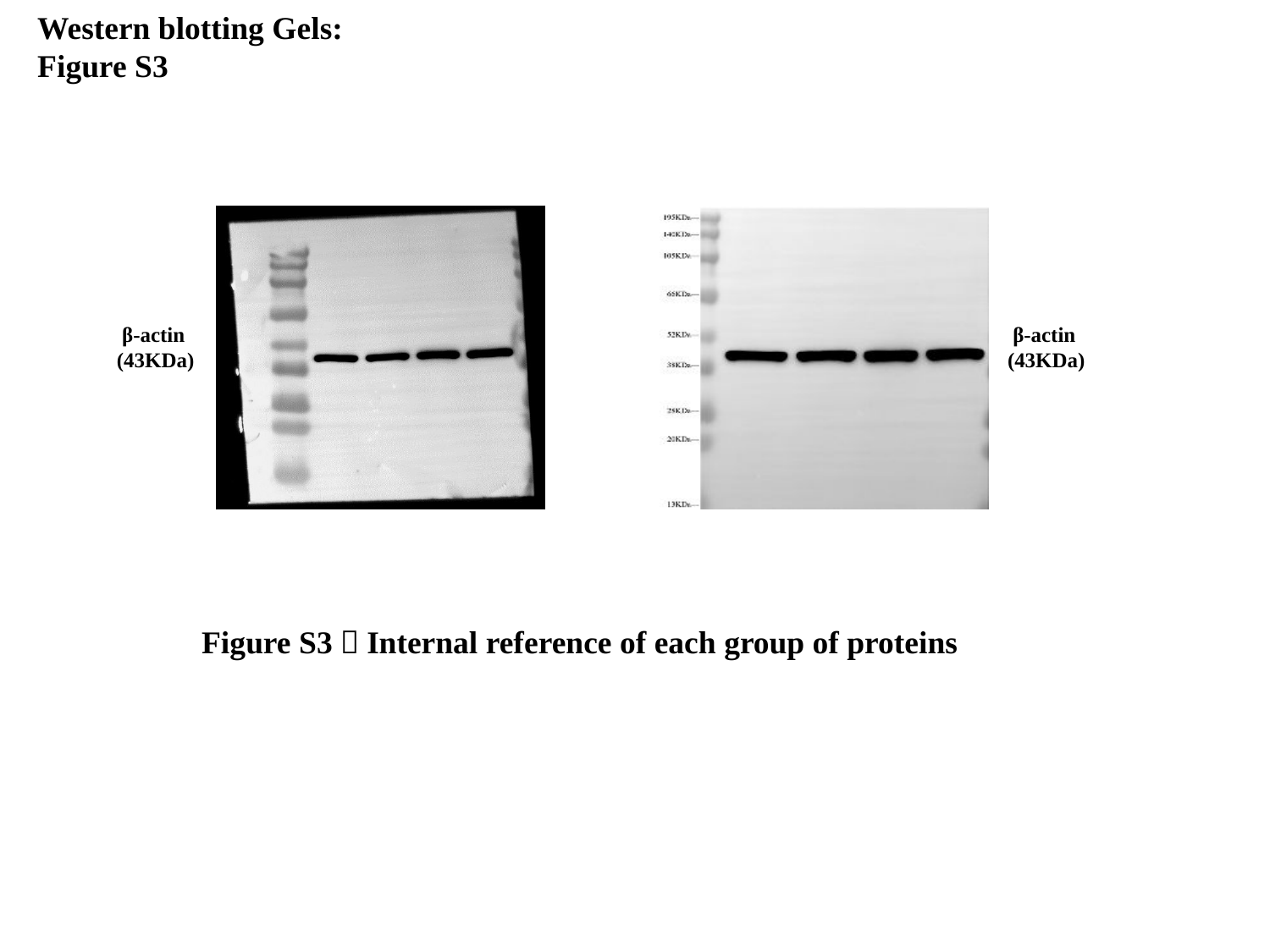

Western blotting Gels:
Figure S3
 β-actin
(43KDa)
 β-actin
(43KDa)
Figure S3：Internal reference of each group of proteins
